# Expression Profile and Potential Functions of Circulating Long Noncoding RNAs in Acute Ischemic Stroke in the Southern Chinese Han Population

**DOI:** 10.3389/fnmol.2019.00290

**Published:** 2019-11-29

**Authors:** Shenghua Li, Huilei Zheng, Lan Chen, Chen Xu, Xiang Qu, Zhenxiu Qin, Jinggui Gao, Jinpin Li, Jingli Liu

**Affiliations:** ^1^Department of Neurology, The First Affiliated Hospital of Guangxi Medical University, Nanning, China; ^2^Department of Medical Examination and Health Management, The First Affiliated Hospital of Guangxi Medical University, Nanning, China; ^3^Department of Internal Medicine, The Second Affiliated Hospital of Guangxi Medical University, Nanning, China

**Keywords:** AIS, lncRNA, circulating blood, RNA-Seq, lncRNA-AC136007.2, lncRNA-C14orf64, Han population

## Abstract

**Background**: Long noncoding RNAs (lncRNAs) have been confirmed to be associated with ischemic stroke (IS); however, their involvement still needs to be extensively explored. Therefore, we aimed to study the expression profile of lncRNAs and the potential roles and mechanisms of lncRNAs in the pathogenesis of acute ischemic stroke (AIS) in the Southern Chinese Han population.

**Methods**: In this study, lncRNA and mRNA expression profiles in AIS were analyzed using high-throughput RNA sequencing (RNA-Seq) and validated using quantitative real-time polymerase chain reaction (qRT-PCR). Gene Ontology (GO), Kyoto Encyclopedia of Genes and Genomes (KEGG) pathway enrichment and network analyses were performed to predict the functions and interactions of the aberrantly expressed genes. Receiver operating characteristic (ROC) curve analysis was performed to evaluate the diagnostic value of lncRNAs in AIS.

**Results**: RNA-Seq analysis showed that 428 lncRNAs and 957 mRNAs were significantly upregulated, while 791 lncRNAs and 4,263 mRNAs were downregulated in patients with AIS when compared with healthy controls. GO enrichment and KEGG pathway analyses of differentially expressed genes showed that the apoptosis, inflammatory, oxidative and calcium signaling pathways were potentially implicated in AIS pathology. The PCR results showed that the selected lncRNA-C14orf64 and lncRNA-AC136007.2 were significantly downregulated in AIS. ROC curve analysis showed that the area under the ROC curve (AUC) values of lncRNA-C14orf64 and lncRNA-AC136007.2 between AIS and healthy controls were 0.74 and 0.94, respectively.

**Conclusion**: This study provides evidence of altered expression of lncRNAs and their potential functions in AIS. Our findings may facilitate pathological mechanistic studies of lncRNAs in AIS and provide potential diagnostic biomarkers and therapeutic targets for AIS.

## Introduction

Stroke is one of the leading causes of death and the most common cause of serious long-term disability in adults worldwide (Liu et al., 2011; Writing Group Members et al., 2016). As the most common type of stroke, ischemic stroke (IS) accounts for over 85% of all strokes (van der Worp and van Gijn, 2007). Currently, the only approved treatments for acute ischemic stroke (AIS) are intravenous thrombolysis with intravenous tissue plasminogen activator (t-PA) within 3–4.5 h after symptom onset and mechanical thrombectomy (Berkhemer et al., 2015; Derex and Cho, 2017). However, the narrow 3–4.5 h therapeutic time window for rt-PA thrombolysis urges researchers to seek new treatment strategies and medicines. Although substantial progress has been made in fully understanding the pathomechanism of neuronal death caused by stroke in recent years, clinical methods to effectively reverse neuronal damage caused by ischemia are still lacking. Therefore, further understanding the pathophysiological mechanism of the development and recovery of AIS might provide a new and more effective way to improve outcomes after AIS.

According to the human Encyclopedia of DNA Elements (ENCODE) project, approximately 80% of the Homo sapien genome is transcribed as nonprotein-coding RNAs (ncRNAs; ENCODE Project Consortium, 2012). Generally, ncRNAs, including long noncoding RNAs (lncRNAs) and microRNAs, are abundantly expressed in the human central nervous system and are implicated in several neurological diseases (Cao et al., 2006; Qureshi et al., 2010). LncRNAs represent a class of transcripts longer than 200 nucleotides in length that lack protein-coding capability and are considered “junk” DNA. However, with the rapid development of RNA sequencing (RNA-Seq) technology, current studies have demonstrated that lncRNAs play a pivotal role in regulating gene expression (Martianov et al., 2007) and are involved in many critical biological processes, such as differentiation, organogenesis, apoptosis, genomic imprinting, regulation of mRNA splicing and translational control (Mercer et al., 2009; Geisler and Coller, 2013; Ulitsky and Bartel, 2013). In recent years, numerous researchers have shown great interest in exploring the association between lncRNA profiles and neurological disorders (Wu et al., 2013; Dykstra-Aiello et al., 2016; He et al., 2018). Importantly, an increasing number of studies have shown that lncRNAs play crucial roles in the pathogenesis of IS (Dykstra-Aiello et al., 2016; Liu et al., 2019). Although the functions of most lncRNAs in neuroprotection and IS remain to be clarified (Dharap et al., 2012), RNA-Seq analyses have indicated that the abnormal expression of lncRNAs is closely associated with IS, and increasing evidence has confirmed that lncRNAs generally contribute to the development and progression of IS as well as to recovery from IS (Bhattarai et al., 2017; Guo et al., 2017; Wang et al., 2017; Zhang et al., 2018; Liu et al., 2019).

Currently, the functional study of lncRNAs is mainly focused on animal models of IS. As distinct differences exist between animals and humans, the lncRNA profiles in AIS definitely need to be further investigated, especially among different races. Moreover, current research on this issue has focused on only a small number of lncRNAs, such as MIAT, GAS5, MALAT1, SNHG1 and ANRIL (Guo et al., 2017; Chen et al., 2018; Zhang et al., 2018; Zhu et al., 2018; Liu et al., 2019). Many lncRNAs have not yet been discovered in patients with AIS; more importantly, the functions of most lncRNAs have not yet been clarified (Kopp and Mendell, 2018). Therefore, we aim to use the RNA-Seq technique to generate an expression profile of lncRNAs and mRNAs related to AIS from the circulating blood of Southern Chinese Han patients and elaborate the potential function of lncRNAs *via* interactions with specific mRNAs.

To the best of our knowledge, this is the first study to investigate the lncRNA expression profile in AIS in the South Chinese Han population. In the present study, the lncRNA expression profile in the circulating blood of patients with AIS was investigated by using RNA-Seq analysis, and the results were validated with real-time polymerase chain reaction (RT-PCR). Moreover, bioinformatics analyses were further applied to predict the functions of differentially expressed lncRNAs. Furthermore, the effects and potential underlying molecular mechanisms of lncRNAs on the pathological process of AIS were elaborated through interactions with specific mRNAs. These findings will help illustrate the molecular pathological mechanism of AIS and identify new biomarkers and therapeutic targets for AIS in the future.

## Materials and Methods

### Study Subjects

This study was approved by the Ethical Committee of The First Affiliated Hospital of Guangxi Medical University (no. 2018-KY-E-063). Thirty-two patients who received a clinical diagnosis of AIS involving large vessel occlusion of the anterior circulation between September 2018 and June 2019 were selected for inclusion in this study. Thirty-two healthy controls from the Physical Examination Center of The First Affiliated Hospital of Guangxi Medical University from September 2018 to November 2018 were also included. Three AIS samples and three control samples were randomly selected from 32 patients and 32 healthy controls for RNA-seq, and all the samples were used for the validation of differentially expressed lncRNAs. Written consent forms were obtained from all participants or their next of kin before sample collection. The inclusion criteria for patients with AIS were as follows: (1) patients were men and women aged 50 and over; (2) patients were of Southern Chinese Han ethnicity; (3) patients had available blood samples; (4) AIS was confirmed by brain imaging (CT/MRI); (5) IS was caused by large artery occlusion occurring in the anterior circulation; (6) patients did not undergo recanalization therapy; (7) patients had regular physical examinations; (8) patients had no history of cardiovascular or cerebrovascular diseases; and (9) patients had no history of severe lung, liver, kidney or other systemic diseases.

Patients with AIS were excluded if they met any of the following qualifications: (1) they had cerebral hemorrhage confirmed by skull CT; (2) they underwent endovascular intervention therapy; (3) they had severe liver or kidney dysfunction; (4) they required diuretics; (5) they had a history of stroke; (6) they had autoimmune disorders or hematological diseases; (7) they had brain tumors or other forms of cancer; and (8) they participated in an ongoing clinical trial study. Healthy controls with a history of cardiovascular or cerebrovascular disorders, hematologic disease, recent infection or other medical illnesses were excluded.

The historical assessments and physical examinations for all the included participants were performed by a neurology resident. The categories of stroke etiologies were assessed using the Trial of ORG 10172 in Acute Stroke Treatment (TOAST) criteria (Adams et al., 1993) and classified as large vessel atherosclerosis, small vessel occlusion, cardioembolism, stroke with determined etiology or stroke with undetermined etiology. Patients with large vessel atherosclerotic stroke were included in this study. The classifications of IS subtypes according to the Oxfordshire Community Stroke Project (OCSP) criteria (Bamford et al., 1991) were as follows: total anterior circulation infarct (TACI), partial anterior circulation infarct (PACI), posterior circulation infarct (POCI), and lacunar infarct (LACI).

Demographic and clinical information, including age, sex, hypertension, hyperlipidemia, diabetes mellitus and homocysteine, were collected.

### Sample Collection and Total RNA Extraction

Circulating blood samples were collected in sterile evacuated tubes containing EDTA by venipuncture on the day of admission or the next day and processed immediately or within a few hours. Total RNA was extracted from the circulating blood of AIS patients and healthy controls using TRIzol reagent (Life Technologies, Franklin, MA, USA) according to the manufacturer’s instructions. Then, the RNA concentration and purity were analyzed by a NanoDrop 2000 spectrophotometer (Thermo Fisher Scientific, Waltham, MA, USA). RNA contamination and degradation were assessed by electrophoresis on 1% agarose gels. RNA integrity and quality were evaluated by an Agilent 2100 Bioanalyzer (Agilent Technologies, La Jolla, CA, USA). All total RNA samples were stored at −80°C until further analysis.

### Construction of RNA-Seq Libraries

Transcriptome sequencing was performed by Beijing Novogene Bioinformatics Technology Co., Limited (Beijing, China^1^). RNA-Seq libraries were generated according to the manufacturer’s protocols using the NEB Next Ultra Directional RNA Library Prep Kit for Illumina (Illumina, CA, USA). The gene expression profiles of the AIS and healthy control groups were investigated using Illumina HiSeq™ 2500 Sequencing according to the manufacturer’s guide (Illumina, CA, USA). The raw data were normalized. Clean reads were obtained from the raw data by discarding adapter and poly-N sequences and reads of low quality. All further downstream analyses were based on the high-quality clean data. Altered lncRNAs and mRNAs with statistically significant differences between healthy controls and patients with AIS were determined by *P*-value, false discovery rate (FDR) and fold-change filtering. Transcripts with a *P*-value of ≤0.05 and threshold values of ≥2-fold change were specified as differentially expressed transcripts.

### GO and KEGG Pathway Analyses

GO enrichment analysis was performed to annotate the functions of the differentially expressed mRNAs and lncRNAs with GO terms. GO enrichment analysis describes the characteristics of altered genes from three structured networks of terms: biological processes, cellular components and molecular functions. The abnormal expression of mRNAs between AIS patients and healthy controls was included in GO enrichment and Kyoto Encyclopedia of Genes and Genomes (KEGG) pathway analyses according to the latest KEGG database. The above analysis enabled us to determine the biological pathways of the altered mRNAs in the acquired samples.

### LncRNA-mRNA Coexpression Analysis

LncRNA-mRNA networks were constructed according to the correlation analysis of differentially expressed mRNAs and lncRNAs. For each mRNA-lncRNA pair, the Pearson correlation was calculated, and significantly correlated pairs with a Pearson correlation coefficient value over 0.98 were selected to construct the network using Cytoscape 3.5.1^2^. We mainly selected genes potentially related to IS to construct the lncRNA-mRNA networks.

### Construction and Analysis of the lncRNA-miRNA-mRNA Network

A competing endogenous RNA (ceRNA) network was constructed by the abnormal expression of lncRNAs, miRNAs and mRNAs. First, lncRNA-mRNA interactions were predicted by correlation analysis of RNA-Seq coexpression to reduce the false positives of paired lncRNA-mRNAs. The Pearson correlation coefficient was used to filter the significant paired mRNAs-lncRNAs from the RNA-Seq data. Only interactions with a Pearson correlation coefficient over 0.98 and a *P*-value <0.01 were included. Next, lncRNA-miRNA interactions were predicted by the LncBook BIG Data Center^3^, and miRNA-mRNA interactions were predicted by TargetScan^4^. These predicted target genes were compared with the differentially expressed mRNAs and lncRNAs detected from RNA-Seq, and the overlapping RNAs were selected for further analysis. Then, the shared pairs from the predicted miRNA-mRNA and miRNA-lncRNA pairs potentially related to IS were selected to build the network. Finally, a miRNA-lncRNA-mRNA network potentially associated with AIS was drawn by Cytoscape based on the selected shared miRNA-lncRNA, lncRNA-mRNA and miRNA-mRNA pairs.

### qRT-PCR Assay

To further validate the reliability of the RNA-Seq results, quantitative real-time PCR (qRT-PCR) assays were performed. The concentration of total RNA in all samples was higher than 400 μg/μl, and the ratio of A260/280 was in the range of 1.8–2.0. Two micrograms of total RNA were reverse transcribed into cDNA using the Thermo Scientific RevertAid First Strand cDNA Synthesis Kit (Thermo Fisher Scientific, Waltham, MA, USA), and RT-PCR was carried out using SYBR Premix Ex Taq (Takara, Dalian, China) according to the standard protocol. LncRNA primers were synthesized by Sangon Biotech (Shanghai, China). GAPDH was used as the internal control. The lncRNA PCR results were quantified using the 2−ΔΔCt method. All experimental reactions were performed in triplicate.

### Statistical Analysis

Prior to statistical analysis, all the data were tested for normality of variance among the different groups through a Kolmogorov–Smirnov test. Comparisons of two groups of continuous variables were performed by using student’s *t*-test, Wilcoxon signed-ranks or Mann–Whitney *U* tests. Statistical analyses of categorical variables were performed using the chi-square test or Fisher’s exact test (Supplementary Table S1). The diagnostic performance of candidate biomarkers was analyzed by receiver operating characteristic (ROC) curves and area under curve (AUC) values. All statistical analyses were performed with GraphPad Prism 7.0 (GraphPad Software, La Jolla, CA, USA) and SPSS version 22.0 (IBM Corporation, Armonk, NY, USA). *P*-values less than 0.05 were considered statistically significant. All data are presented as the mean ± standard error of measurement (SEM). All experiments were performed in triplicate.

## Results

### Demographic Data of Healthy Controls and AIS Patients

The demographic and baseline characteristics of the 32 healthy controls and 32 patients with AIS enrolled in this study from September 2018 to June 2019 were collected. The demographic and clinical features of the participants are shown in Table 1 (Supplementary Table S4). No significant differences in the sex, age or ethnicities or in the incidence rates of diabetes mellitus and hyperlipidemia existed between the patients with AIS and healthy controls (Table 1). However, the frequencies of hypertension and high homocysteine levels were significantly different between patients with AIS and healthy controls (Table 1, *P* < 0.05).

**Table 1:** Clinical characteristics of the studied subjects.

Characteristics	Healthy controls (*n* = 32)	AIS patients (*n* = 32)	*P*-value
Age (years, mean ± SD)	63.75 ± 6.24	64.63 ± 8.03	0.628
Gender (male, %)	17 (53.13%)	18 (56.25%)	0.802
Ethnicity (Han, %)	32 (100%)	32 (100%)	1
Hypertension (n, %)	2 (6.25%)	25 (78.13%)	0.0001
Diabetes mellitus (n, %)	2 (6.25%)	3 (9.74%)	0.64
Hyperlipidemia (n, %)	9 (28.13%)	12 (37.5%)	0.424
Homocysteine (μmol/l, mean ± SD)	12.93 ± 2.79	16.26 ± 5.34	0.02

*AIS, acute ischemic stroke; SD, standard deviation. *P* < 0.05 was considered statistically significant. Hypertension refers to a systolic blood pressure of ≥140 mmHg or a diastolic blood pressure of ≥90 mmHg or the use of antihypertensive agents. Diabetes mellitus was defined as symptoms of diabetes and a fasting blood sugar level >7.0 mmol/l, a random blood glucose level >11.1 mmol/l, a 2 h post prandial blood glucose level >11.1 mmol/l or a prior diagnosis of diabetes. Hyperlipidemia was defined as a cholesterol level >5.72 mmol/l and/or a triglyceride level >1.70 mmol/l or the use of antihyperlipidemic agents*.

### Overview of the lncRNA and mRNA Expression Profiles in Patients With AIS and Healthy Controls

To investigate the pivotal role of lncRNAs and mRNAs that are related to the occurrence and development of AIS, RNA-Seq was carried out to detect the abnormal expression of lncRNAs and mRNAs between AIS patients and healthy controls. In total, 1,219 lncRNAs and 5,220 mRNAs were observed to be significantly differentially expressed between patients with AIS and healthy control subjects. Of those RNAs, 428 lncRNAs and 957 mRNAs were significantly upregulated, while 791 lncRNAs and 4,263 mRNAs were downregulated in patients with AIS compared with the healthy controls (*P* < 0.05, even *q* < 0.05 for mRNA; Supplementary Tables S2, S3).

XLOC_000410 (log2 fold change: 14.16) and XLOC_072127 (log2 fold change: −11.78) were the most upregulated and downregulated lncRNAs, respectively, in patients with AIS compared with healthy controls. Regarding mRNAs, WRNIP1 (log2 fold change: 19.26) and MKNK1 (log2 fold change: −24.93) were the most upregulated and downregulated mRNAs, respectively, in AIS patients compared with healthy controls. Detailed information, including the top 10 dysregulated (over- and underexpressed) known lncRNAs, unknown lncRNAs and mRNAs, is shown in Tables s 2–4, respectively.

**Table 2:** Top 10 upregulated and downregulated known lncRNAs (patients with AIS vs. healthy controls).

Transcript-id	Gene-id	Gene-name	Log2 FC	*P*-value	Up/Down
ENST00000579527.5	ENSG00000265148.5	RP5-1171I10.4	6.45	2.52E-07	up
ENST00000644706.1	ENSG00000285498.1	CTD-2643I7.6	3.84	3.88E-05	up
ENST00000635107.1	ENSG00000282907.1	LA16c-407A10.3	3.81	0.000246	up
ENST00000444042.2	ENSG00000225506.2	RP1-18D14.3	3.53	0.001337	up
ENST00000599993.5	ENSG00000268119.5	CTD-2561J22.5	3.22	0.008346	up
ENST00000453100.1	ENSG00000224177.6	AC099344.4	3.14	0.000446	up
ENST00000526554.1	ENSG00000255477.1	RP11-63D14.1	3.12	0.018556	up
ENST00000540299.1	ENSG00000249790.2	RP11-20D14.6	3.00	4.69E-05	up
ENST00000505731.5	ENSG00000249673.6	RP11-520M5.5	2.95	0.000321	up
LENST00000585593.1	ENSG00000267122.1	AC004490.1	2.87	0.014017	up
ENST00000419542.3	ENSG00000179818.13	AC136007.2	−10.84	3.24E-05	down
ENST00000437964.5	ENSG00000230487.7	AC093734.11	−9.73	0.007396	down
ENST00000620124.1	ENSG00000273951.1	RP4-620E11.8	−9.73	0.010910	down
ENST00000527128.5	ENSG00000254876.5	RP11-23J9.5	−9.58	0.001847	down
ENST00000623001.3	ENSG00000203875.10	SNHG5	−9.53	0.008159	down
ENST00000612725.1	ENSG00000278740.1	RP11-147L13.14	−8.91	0.004420	down
ENST00000609166.1	ENSG00000272979.1	RP11-647K16.1	−8.42	0.038446	down
ENST00000502187.5	ENSG00000246223.8	C14orf64	−7.83	0.000608	down
ENST00000581917.1	ENSG00000264895.1	RP11-421E14.2	−7.70	0.001393	down
ENST00000623493.1	ENSG00000279059.1	RP11-257O5.2	−7.70	0.005771	down

*AIS, acute ischemic stroke; FC, fold change*.

**Table 3:** Top 10 upregulated and downregulated unknown lncRNAs (patients with AIS vs. healthy controls).

Transcript-id	Gene-id	Gene-name	Log2 FC	*P*-value	Up/Down
LNC_000081	XLOC_000410	NA	14.16	6.61E-05	up
LNC_000035	XLOC_000181	NA	12.51	3.50E-07	up
LNC_000059	XLOC_000410	NA	12.50	0.000120	up
LNC_000058	XLOC_000410	NA	10.22	0.000501	up
LNC_000098	XLOC_000410	NA	10.05	7.29E-06	up
LNC_000018	XLOC_000181	NA	8.38	0.000595	up
LNC_003540	XLOC_100197	NA	8.31	0.000171	up
LNC_001081	XLOC_025991	NA	8.27	0.037686	up
LNC_002846	XLOC_079106	NA	7.23	0.00653	up
LNC_004273	XLOC_123308	NA	7.09	0.000385	up
LNC_002619	XLOC_072127	NA	−11.78	2.29E-05	down
LNC_002376	XLOC_064601	NA	−9.88	0.012274	down
LNC_001004	XLOC_024298	NA	−9.64	0.0016751	down
LNC_002936	XLOC_081255	NA	−8.72	0.001630	down
LNC_002114	XLOC_056683	NA	−8.33	0.000992	down
LNC_004552	XLOC_133139	NA	−7.79	4.97E-05	down
LNC_002926	XLOC_081252	NA	−7.76	0.003041	down
LNC_000594	XLOC_012509	NA	−7.67	0.0012667	down
LNC_002930	XLOC_081252	NA	−7.58	0.0261286	down
LNC_003476	XLOC_098284	NA	−7.57	0.0027701	down

*AIS, acute ischemic stroke; FC, fold change*.

**Table 4:** Top 10 upregulated and downregulated mRNAs (patients with AIS vs. healthy controls).

Transcript-id	Gene-id	Gene-name	Log2.FC	*P*-value	Up/Down
ENST00000380764	ENSG00000124535	WRNIP1	19.26	0.00056	up
ENST00000409556	ENSG00000115548	KDM3A	16.86	0.00014	up
ENST00000405977	ENSG00000132640	BTBD3	16.75	0.00166	up
ENST00000616058	ENSG00000094975	SUCO	16.62	8.28E-12	up
ENST00000434687	ENSG00000030419	IKZF2	15.41	0.00046	up
ENST00000593002	ENSG00000132471	WBP2	14.70	1.69E-07	up
ENST00000427614	ENSG00000185347	TEDC1	13.85	0.039518	up
ENST00000620467	ENSG00000104973	MED25	13.51	8.99E-05	up
ENST00000224756	ENSG00000107771	CCSER2	13.39	0.000136	up
ENST00000271139	ENSG00000142961	MOB3C	13.08	0.042181	up
ENST00000553930	ENSG00000100445	SDR39U1	12.72	0.004969	up
ENST00000371945	ENSG00000079277	MKNK1	−24.93	9.62E-10	down
ENST00000216780	ENSG00000100889	PCK2	−20.75	1.57E-05	down
ENST00000574428	ENSG00000123472	ATPAF1	−20.54	0.023406	down
ENST00000612687	ENSG00000149428	HYOU1	−19.84	1.64E-08	down
ENST00000367446	ENSG00000116747	TROVE2	−19.41	0.022855	down
ENST00000225430	ENSG00000108298	RPL19	−19.33	4.36E-15	down
ENST00000628018	ENSG00000126247	CAPNS1	−18.01	0.004723	down
ENST00000417294	ENSG00000071054	MAP4K4	−16.89	0.000334	down
ENST00000547303	ENSG00000175197	DDIT3	−16.71	0.00664	down
ENST00000650508	ENSG00000079277	MKNK1	−16.63	0.012937	down

*AIS, acute ischemic stroke; FC, fold change*.

**Figure 1:**
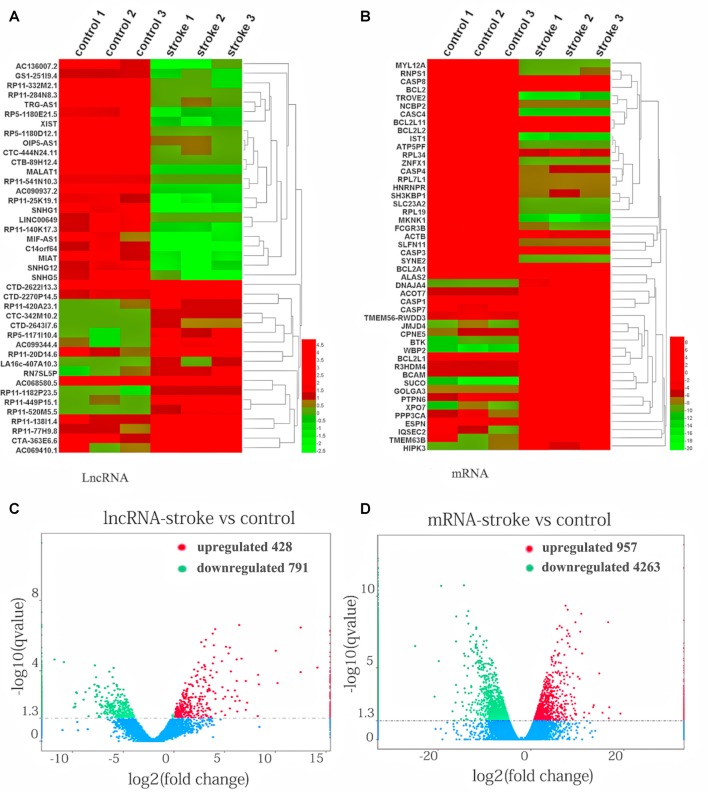
Aberrantly expressed lncRNAs and mRNAs in patients with acute ischemic stroke (AIS) and healthy controls. Heatmap showing the hierarchical clustering of differentially expressed lncRNAs **(A)** and mRNAs **(B)** in AIS patients and healthy controls. Up- and downregulated lncRNAs and mRNAs are represented in red and green, respectively. The volcano plot of lncRNA **(C)** and mRNA **(D)** expression signals in AIS patients and healthy controls. Red circles represent upregulated genes, green circles represent downregulated genes, and the dotted line in the volcano plot represents the default significant fold change.

The expression patterns of lncRNAs (Figure 1A) and mRNAs (Figure 1B) between AIS patients and healthy controls were distinguished *via* hierarchical clustering heatmaps. Red represents high relative expression, and green represents low relative expression. Volcano plots were constructed to evaluate the variation and reproducibility of lncRNA (Figure 1C) and mRNA (Figure 1D) expression in blood between AIS patients and healthy controls. Red circles represent upregulated genes, while green circles represent downregulated genes.

In line with our results, mRNAs such as Bcl2 (Iwata et al., 2010), Bcl-W (Minami et al., 2000), Bcl-XL (Wiessner et al., 1999) and Casp3 (Gorup et al., 2015) and some miRNAs, such as miR-143-3p (Tiedt et al., 2017), miR-26b-3p (Yuan et al., 2016) and miR-339-5p (Dhiraj et al., 2013), that were previously reported to be up- or down-regulated in AIS patients were also found in this study.

Well-known lncRNAs, such as MALAT1 (Li et al., 2017), SNHG1 (Zhang et al., 2018), MIAT (Zhu et al., 2018) and SNHG12 (Zhao et al., 2018), that were previously reported as diagnostic and prognostic biomarker molecules of IS were also observed among the dysregulated lncRNAs.

### GO and KEGG Pathway Analyses

**Figure 2:**
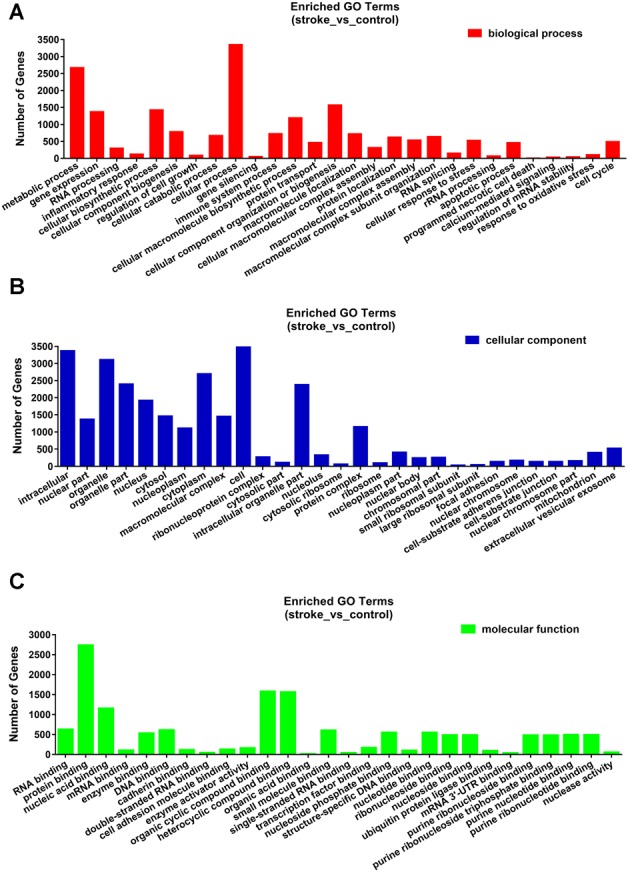
Gene ontology (GO) enrichment analysis of differentially expressed genes in AIS. **(A)** Biological process. **(B)** Cellular component. **(C)** Molecular function.

**Figure 3:**
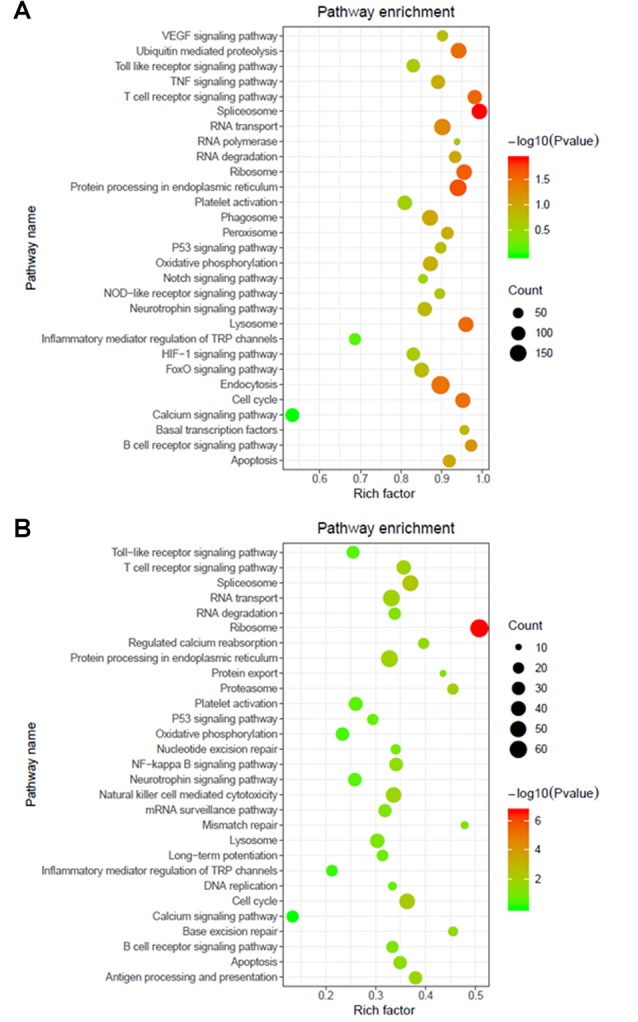
Pathway analysis of differentially expressed RNAs. **(A)** GO and Kyoto Encyclopedia of Genes and Genomes (KEGG) pathway analyses of potential genes associated with differentially expressed lncRNAs. **(B)** GO and KEGG pathway enrichment analyses of differentially expressed mRNAs. The Rich factor represents the degree of enrichment. The node size shows the number of selected genes, and the color scale represents the –log (*P*-value).

The functions and mechanisms of most lncRNAs remain elusive and may be determined from their related protein-coding genes. Therefore, we analyzed the GO and KEGG pathway enrichments of the differentially expressed mRNAs and lncRNAs between patients with AIS and healthy controls. We observed that cellular process (GO: biological process), metabolic process (GO: biological process), cell (GO: cellular component), protein binding (GO: molecular function), intracellular (GO: cellular component) and enzyme activator activity (GO: molecular function) were the most enriched terms (Figure 2). The significantly enriched GO terms also included apoptotic process (GO:0006915), inflammatory response (GO:0006954), response to oxidative stress (GO:0006979), calcium-mediated signaling (GO:0019722), and response to stress (GO:0006950), which may be closely related to the pathophysiology of AIS; the detailed results are presented in Figure 2. Correlation analysis of the expression levels of lncRNAs and nearby mRNAs revealed that some lncRNAs were associated with nearby mRNAs. GO enrichment and KEGG pathway analyses of these mRNAs implied that they are involved in numerous biological processes, such as apoptosis, ribosomes, the cell cycle, P53, RNA transport, vascular growth, endocytosis, oxidative response and the inflammatory response signaling pathway. Pathway analysis of the significantly differentially expressed lncRNAs related to differentially expressed mRNAs identified significantly enriched KEGG pathways (Figure 3A). The significantly differentially expressed lncRNAs were enriched in pathways such as the ribosome (hsa03010), spliceosome (hsa03040), RNA transport (hsa03013), cell cycle (hsa04110), apoptosis (hsa04210), inflammatory mediator regulation of transient receptor potential (TRP) channels (hsa04750), oxidative phosphorylation (hsa00190), calcium signaling (hsa04020), p53 signaling (hsa04115) and Toll-like receptor signaling (hsa04620). Moreover, because we speculated that abnormally expressed mRNAs may also be involved in these signaling pathways, we generated a bubble diagram to identify the signaling pathways. The results showed that bubbles among the ribosome, cell cycle, P53, RNA transport, apoptosis, platelet activation, calcium, Toll-like receptor, and inflammatory signaling pathways were identified to a high degree (Figure 3B). The detailed results of the pathway enrichment analysis are shown in Figure 3.

### Validation of the lncRNA Expression Profile and Identification of Novel lncRNA Biomarkers for AIS

**Figure 4:**
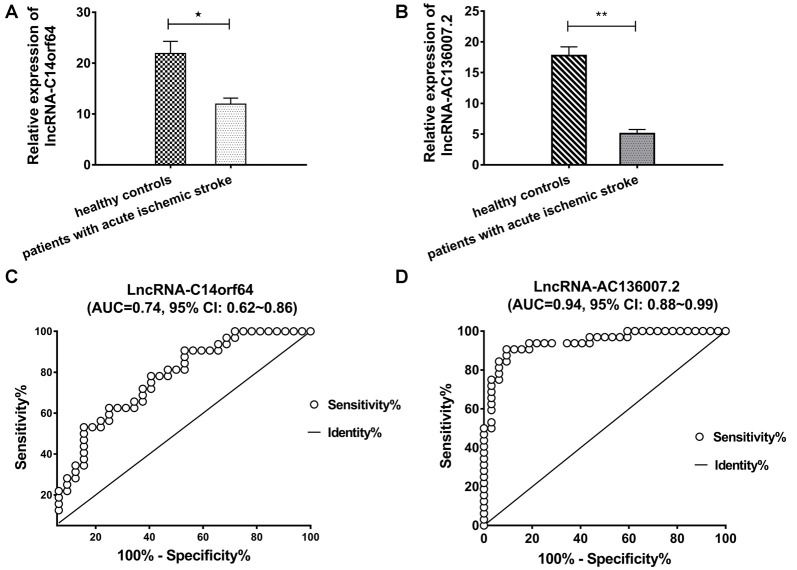
Real-time polymerase chain reaction (RT-PCR) analysis of lncRNA expression and receiver operating characteristic (ROC) curves measuring the diagnostic efficiency of selected lncRNAs. **(A)** RT-PCR validation of the relative expression levels of lncRNA-C14orf64. **(B)** Relative expression levels of lncRNA-AC136007.2. **(C)** ROC curve for discriminating patients with AIS from healthy controls by lncRNA-C14orf64 had an area under curve (AUC) of 0.74 (95% CI, 0.62–0.86, sensitivity 0.63, specificity 0.75). **(D)** The ROC curve for discriminating patients with AIS from healthy controls *via* lncRNA-AC136007.2 had offered the greater diagnostic ability with an AUC of 0.94 (95% CI, 0.88–0.99, sensitivity 90.63, specificity 90.63). The measured RNA expression levels were normalized by GAPDH (*P < 0.05, **P < 0.001).

Among the top 10 most up- and downregulated known lncRNAs, correlation analysis of RNA-Seq coexpression data between lncRNAs and nearby mRNAs demonstrated that only lncRNA-AC136007.2 and lncRNA-C14orf64 correlated with nearby mRNAs. These two lncRNAs were selected for qRT-PCR and verified in 32 AIS patients and 32 healthy controls. Both of these lncRNAs were significantly differentially expressed between AIS patients and healthy controls and demonstrated the same directional trends as those observed in the RNA-Seq data, indicating a strong consistency between the RNA-Seq data and the qRT-PCR results. The PCR results showed that lncRNA-C14orf64 and lncRNA-AC136007.2 were significantly downregulated in the circulating blood of AIS patients (Figures 4A,B; Supplementary Tables S5–S7). To evaluate the potential diagnostic value of lncRNAs in AIS, we carried out ROC curve analysis to evaluate the ability of lncRNA-C14orf64 and lncRNA-AC136007.2 to distinguish patients with AIS from healthy controls. ROC curve analysis showed that the AUC values of lncRNA-C14orf64 and lncRNA-AC136007.2 between AIS patients and healthy controls were 0.74 (95% CI: 0.62–0.86, sensitivity 0.63, specificity 0.75) and 0.94 (95% CI: 0.88–0.99, sensitivity 90.63, specificity 90.63), respectively (Figures 4C,D). Thus, lncRNA-C14orf64 and lncRNA-AC136007.2 were deemed considerably valuable for the diagnosis of patients with AIS. Furthermore, lncRNA-C14orf64 was predicted to target genes related to AIS, such as Bcl2, Bcl-XL, Bcl-W, BCL2L13, BCL2L14 and BCLAF1, which are critical in the apoptotic process. Based on the *P*-value, fold change, predicted target genes potentially associated with AIS, and a series of filtering pipelines, lncRNA-C14orf64 was selected for further investigation of its effects and mechanisms in AIS.

### Construction of a lncRNA-mRNA Coexpression Network

**Figure 5:**
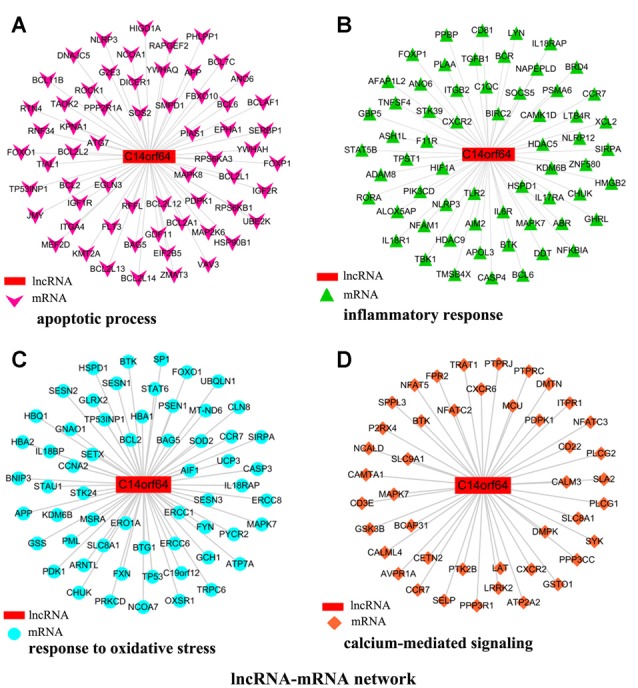
Network of genes correlated with lncRNA-C14orf64. LncRNAs are shown as red rectangular nodes, and genes associated with apoptosis are shown as pink arrow nodes **(A)**; genes associated with the inflammatory response are shown as green triangle nodes **(B)**; genes associated with response to oxidative stress are shown as blue circular nodes **(C)**; and genes associated with calcium-mediated signaling are shown as brown square nodes **(D)**.

To investigate the association between lncRNA-C14orf64 and mRNAs, a lncRNA-mRNA coexpression network was constructed based on the correlation analysis, and only the lncRNA/mRNA gene pairs with a Pearson correlation coefficient over 0.98 and a *P*-value < 0.05 were included. Current studies have shown that IS can induce the transcription of genes related to apoptosis, the inflammatory response, oxidative stress and intracellular calcium overload (Gutiérrez et al., 2009). Therefore, the genes we selected were mainly enriched in pathways related to IS, such as the apoptotic process (Figure 5A), inflammatory response (Figure 5B), response to oxidative stress (Figure 5C), and calcium-mediated signaling (Figure 5D). The network of genes correlating with lncRNA-C14orf64 is shown in Figure 5.

### Construction and Analysis of a lncRNA-miRNA-mRNA Network

**Figure 6:**
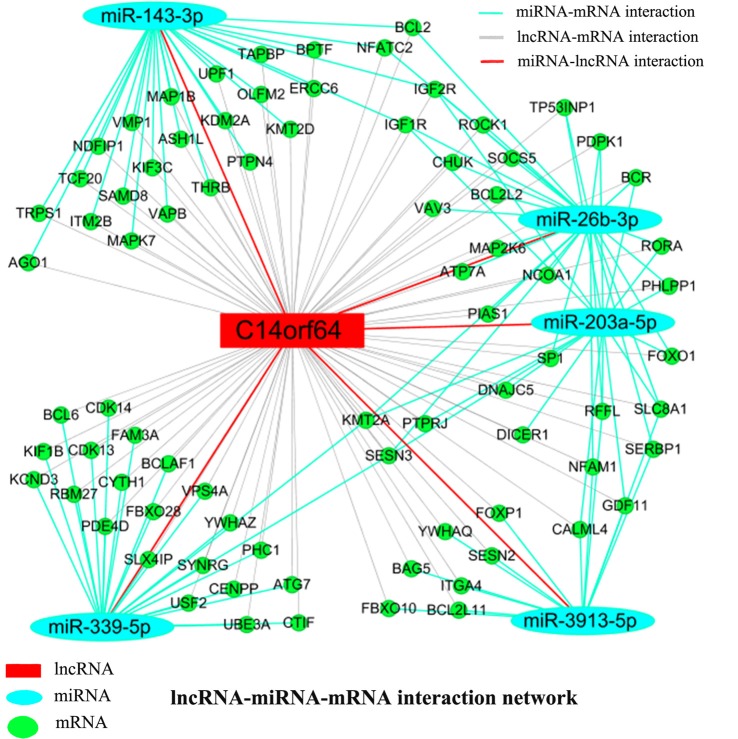
LncRNA-miRNA-mRNA network. A ceRNA network was constructed corresponding to the miRNA, lncRNA and mRNA variations in AIS patients. Light blue oval nodes represent microRNAs, green circular nodes represent mRNAs, and red rectangular nodes represent lncRNAs. Black edges represent the possible relationship between lncRNAs and mRNAs, light blue edges represent the inhibitory effect of miRNAs on mRNAs, and red edges show the relationship between miRNAs and lncRNAs.

Recent studies have shown that mRNAs and lncRNAs with similar sequences can be bound by the same miRNA. LncRNAs may serve as ceRNAs to sponge miRNAs, thus modulating the derepression of miRNA targets and then increasing the expression levels of mRNAs at the posttranscriptional level (Cesana et al., 2011). Pearson correlation coefficients estimated the complexity of interactions among genes neighboring the core gene, which were merged with miRNAs to explore lncRNA, miRNA and mRNA coexpression. Network analysis was performed to investigate which gene(s) may play a crucial role in AIS. A lncRNA-miRNA-mRNA network was constructed from predicted genes that may be associated with AIS. The lncRNA-miRNA-mRNA network indicated the relationships among lncRNAs, miRNAs and mRNAs. Bioinformatics analysis further showed that many genes (such as Bcl2, BCL2L2, BCL2L11, BCL2A1 and KDM2A), lncRNAs (C14orf64) and miRNAs (such as miR-26b-3p, miR-203a-5p, miR-339-5p, miR-3913-5p and miR-143-3p) potentially play a pivotal role in the network. The network analysis results showed that mRNAs can be coregulated by lncRNAs together with different miRNAs and that mRNAs can be coregulated by different miRNAs together with different lncRNAs. For example, KMT2A can be simultaneously regulated by miRNAs (miR-26b-3p, miR-203a-5p and miR-339-5p) and lncRNAs. The results presented here imply the potential regulatory functions of lncRNA-C14orf64 *via* interactions with specific miRNAs and mRNAs, which may play a crucial role in the pathogenesis of AIS (Figure 6).

## Discussion

Given the high prevalence of IS in China, with high morbidity, mortality and disability rates, investigating the genetic mechanisms underlying AIS in the Southern Chinese Han population is very meaningful. Previous studies have demonstrated a set of peripheral blood gene expression changes specific to those with IS exhibiting brain neuronal death compared with those without neuronal death, confirming a close association between blood gene expression and brain damage (Tang et al., 2003). Moreover, circulating leukocytes exhibit cerebral ischemia-induced changes in the RNA expression profile detectable in circulating blood, as demonstrated in a series of animal models of IS (Tang et al., 2001; Sharp et al., 2011; Dagonnier et al., 2018; Li et al., 2019). Although emerging studies have shown that numerous lncRNAs may be involved in the regulation of IS pathophysiologic processes (Dykstra-Aiello et al., 2016; Wu et al., 2017; Zhang et al., 2018; Liu et al., 2019; Zhu et al., 2019), their roles in the pathological mechanisms, nosogenesis and progression of IS still need to be further clarified (Beermann et al., 2016). In the present study, we used high-throughput sequencing technologies to generate a signature profile of numerous lncRNAs and mRNAs in AIS compared with matched healthy controls, and bioinformatics analysis was performed to analyze the data. The RNA-Seq analysis showed that a significant number of lncRNAs and mRNAs are altered in AIS, indicating their potential regulatory functions in the pathophysiological processes of this disease.

First, we screened for genes that were differentially expressed between patients with AIS and healthy controls. The RNA-Seq data showed that 1,219 lncRNAs and 5,220 mRNAs were significantly differentially expressed between patients with AIS and healthy controls. Of these RNAs, 428 lncRNAs and 957 mRNAs were significantly increased, while 791 lncRNAs and 4,263 mRNAs were decreased. Using qRT-PCR, we selected and validated the expression of lncRNA-AC136007.2 and lncRNA-C14orf64 in AIS. The RT-PCR results showed that lncRNA-C14orf64 and lncRNA-AC136007.2 were significantly downregulated in AIS patients. The RT-PCR results were consistent with the RNA-Seq data, demonstrating that the RNA-Seq results were reliable. ROC curve analysis showed that lncRNA-AC136007.2 and lncRNA-C14orf64 can be used as potential diagnostic biomarkers of AIS. Based on a series of filtering pipelines, as mentioned above in the “Results” section, we selected lncRNA-C14orf64 for downstream experiments.

Second, we analyzed the potential functions of significantly differentially expressed lncRNAs *via* interactions with specific mRNAs. A comprehensive analysis of the differential expression pattern of lncRNAs and mRNAs will further our understanding of the biological functions of the abnormally expressed lncRNAs. Therefore, correlation analysis was conducted between the differentially expressed lncRNAs and IS-related protein-coding genes, and the correlations between the transcripts of the two groups were then identified. GO classification, coexpression and KEGG pathway enrichment analyses of the differentially expressed lncRNAs and mRNAs indicated that the apoptotic process, inflammatory response, response to oxidative stress, and calcium-mediated signaling were among the significant biological processes involved in the pathology of AIS. Among these processes, we focused on the apoptotic pathway. Our study analyzed the mRNA expression levels and observed that the expression levels of Bcl2, BCL2L2 and BCL2A1 were significantly lower in patients with AIS than in healthy controls. RNA-Seq showed that the lncRNA coding gene profile displayed concomitant downregulation of lncRNA-C14orf64 and Bcl2, BCL2L2 and BCL2A1 in the blood of AIS patients. Surprisingly, none of the top 10 most up- and downregulated lncRNAs have been related to AIS in previous studies; however, we found positive correlations between lncRNA-C14orf64, which is among the top ten most downregulated lncRNAs in AIS, and Bcl2, BCL2L2 and BCL2A1. BCL2L2 and BCL2A1 belong to the Bcl-2 protein family, the members of which are pivotal regulators of apoptosis. Bcl-2 represents the first known component of the pathophysiological cell death mechanism. Upon further investigation, researchers have found that Bcl-2 belongs to an expanding family of proteins that act as anti- and proapoptotic regulators of cell development and tissue homeostasis. Additionally, Bcl2 can block the proapoptotic actions of other members of the Bcl-2 family, such as Bad and Bax (Merry and Korsmeyer, 1997). The antiapoptotic function of Bcl-2 and Bcl-w is well demonstrated in models of IS, and increased expression levels of Bcl-2 and Bcl-w lead to the prevention of neuronal apoptosis by inhibiting the expression of Bax, which is a critical regulator of mitochondrial fission (Chao and Korsmeyer, 1998; Minami et al., 2000; Mikhailov et al., 2001; Hu et al., 2004).

Finally, we further explored the role of a selected lncRNA (lncRNA-C14orf64) in the pathological process of AIS by constructing a ceRNA network. Because of the complicated dynamic interaction between miRNA-mRNA pairs and miRNA-lncRNA-mRNA combinations, it is generally believed that alterations in the expression patterns of members in each group may concomitantly affect the outcome of their regulation of biological functions in various disorders (Beermann et al., 2016; Li et al., 2016; Chuang and Khorram, 2018). Therefore, we also constructed and analyzed the miRNA-lncRNA-mRNA network based on ceRNAs to reveal the function of lncRNA-C14orf64 in AIS. LncRNA-miRNA interactions were predicted by the LncBook BIG Data Center, revealing that lncRNA-C14orf64 potentially interacts with 82 miRNAs. Among these miRNAs, three miRNAs, miR-143-3p (Tiedt et al., 2017), miR-26b-3p (Yuan et al., 2016) and miR-339-5p (Dhiraj et al., 2013), were reported to be differentially expressed in IS in a previous study. Functionally, these miRNAs may potentially regulate the expression of IS-related protein-coding genes related to angiogenesis, apoptosis, inflammation, immune response, oxidative stress and atherosclerosis, which are characteristics of AIS. GO and KEGG pathway enrichment analyses of the sequencing data regarding abnormally expressed mRNAs showed that abnormally expressed transcripts in AIS are mainly involved in apoptosis, the cell cycle, the proteasome, the proinflammatory response, natural killer cell-mediated cytotoxicity, the T cell receptor signaling pathway, the NF-κB signaling pathway and RNA degradation. Our findings are consistent with the results of previous studies, which suggested that the pathogenic mechanisms underlying secondary damage at the cellular and molecular levels during AIS involve decreased antioxidant abilities and increased oxidative stress, free radical release, excitotoxicity, acute inflammatory response and apoptosis (Ritz et al., 2008; Cai et al., 2015; Hadadha et al., 2015).

The present study does have some limitations. First, the RNA-Seq data are limited to only a small number of samples. The PCR results were obtained from a small sample of only 32 AIS patients and 32 healthy controls, and further experimental investigation of a large sample size is necessary to assess and validate the results. Additionally, most of the conclusions of this study were generated based on only the RNA-Seq data analysis. Furthermore, we merely predicted the functions of abnormal expressed lncRNAs and were unable to validate exactly how these lncRNAs regulate target gene expression. The functional implications of the dysregulated lncRNAs await systematic investigations. In the future, we aim to further investigate and focus on the molecular mechanisms of lncRNAs in the pathological process of AIS.

## Conclusion

Overall, our results provide the differential expression profile of lncRNAs in AIS in the Southern Chinese Han population with concurrent integrated expression of mRNAs and the potential regulatory functions of lncRNAs were elaborated *via* interactions with specific mRNAs which are known to be crucial in the pathological process of AIS. Further study is necessary to explore the fundamental molecular mechanisms and biological functions of lncRNAs in AIS.

## Data Availability Statement

The datasets generated for this study can be found in GEO (accession number: GSE140275; https://www.ncbi.nlm.nih.gov/geo/query/acc.cgi?acc=GSE140275).

## Ethics Statement

The experimental protocol was approved by the Research Ethics Committee of The First Affiliated Hospital of Guangxi Medical University (no. 2018-KY-E-063). The patients/participants provided their written informed consent to participate in this study.

## Author Contributions

SL, JLiu and JLi conceived and supervised the project. SL and HZ designed the experiments. SL, HZ, CX, XQ, ZQ and JG performed all the experiments and collected the data. SL and LC analyzed the data and made the figures. SL, HZ and LC wrote the manuscript.

## Conflict of Interest

The authors declare that the research was conducted in the absence of any commercial or financial relationships that could be construed as a potential conflict of interest.

## Footnotes

^1^www.novogene.com/

^2^https://cytoscape.org

^3^https://bigd.big.ac.cn/lncbook/index

^4^http://www.targetscan.org/vert_72/

## Supplementary Material

TABLE S1 Results of statistical analysis.

TABLE S2 Differentially expressed lncRNAs between AIS patients and healthy controls.

TABLE S3 Differentially expressed mRNAs between AIS patients and healthy controls.

TABLE S4 Demographic data of AIS patients and healthy controls.

TABLE S5 Statistical analysis of lncRNA-C14orf64 qRT-PCR data.

TABLE S6 QRT-PCR data of lncRNA-AC136007.2 and statistical analysis.

TABLE S7 QRT-PCR data of lncRNA-C14orf64.

## References

[B1] Adams H. P. Jr. Bendixen B. H. Kappelle L. J. Biller J. Love B. B. Gordon D. L.. (1993). Classification of subtype of acute ischemic stroke. Definitions for use in a multicenter clinical trial. TOAST. Trial of Org 10172 in Acute Stroke Treatment. Stroke 24, 35–41. 10.1161/01.str.24.1.35 7678184

[B2] Bamford J. Sandercock P. Dennis M. Burn J. Warlow C. (1991). Classification and natural history of clinically identifiable subtypes of cerebral infarction. Lancet 337, 1521–1526. 10.1016/0140-6736(91)93206-o 1675378

[B3] Beermann J. Piccoli M. T. Viereck J. Thum T. (2016). Non-coding RNAs in development and disease: background, mechanisms, and therapeutic approaches. Physiol. Rev. 96, 1297–1325. 10.1152/physrev.00041.2015 27535639

[B4] Berkhemer O. A. Fransen P. S. Beumer D. van den Berg L. A. Lingsma H. F. Yoo A. J.. (2015). A randomized trial of intraarterial treatment for acute ischemic stroke. N. Engl. J. Med. 372, 11–20. 10.1056/NEJMoa1411587 25517348

[B5] Bhattarai S. Pontarelli F. Prendergast E. Dharap A. (2017). Discovery of novel stroke-responsive lncRNAs in the mouse cortex using genome-wide RNA-seq. Neurobiol. Dis. 108, 204–212. 10.1016/j.nbd.2017.08.016 28855129

[B6] Cai J. Cao S. Chen J. Yan F. Chen G. Dai Y. (2015). Progesterone alleviates acute brain injury *via* reducing apoptosis and oxidative stress in a rat experimental subarachnoid hemorrhage model. Neurosci. Lett. 600, 238–243. 10.1016/j.neulet.2015.06.023 26101829

[B7] Cao X. Yeo G. Muotri A. R. Kuwabara T. Gage F. H. (2006). Noncoding RNAs in the mammalian central nervous system. Annu. Rev. Neurosci. 29, 77–103. 10.1146/annurev.neuro.29.051605.112839 16776580

[B8] Cesana M. Cacchiarelli D. Legnini I. Santini T. Sthandier O. Chinappi M.. (2011). A long noncoding RNA controls muscle differentiation by functioning as a competing endogenous RNA. Cell 147, 358–369. 10.1016/j.cell.2011.09.028 22000014 PMC3234495

[B9] Chao D. T. Korsmeyer S. J. (1998). BCL-2 family: regulators of cell death. Annu. Rev. Immunol. 16, 395–419. 10.1146/annurev.immunol.16.1.395 9597135

[B10] Chen F. Zhang L. Wang E. Zhang C. Li X. (2018). LncRNA GAS5 regulates ischemic stroke as a competing endogenous RNA for miR-137 to regulate the Notch1 signaling pathway. Biochem. Biophys. Res. Commun. 496, 184–190. 10.1016/j.bbrc.2018.01.022 29307821

[B11] Chuang T. D. Khorram O. (2018). Expression profiling of lncRNAs, miRNAs, and mRNAs and their differential expression in leiomyoma using next-generation RNA sequencing. Reprod. Sci. 25, 246–255. 10.1177/1933719117711265 28587571

[B13] Dagonnier M. Wilson W. J. Favaloro J. M. Rewell S. S. J. Lockett L. J. Sastra S. A.. (2018). Hyperacute changes in blood mRNA expression profiles of rats after middle cerebral artery occlusion: towards a stroke time signature. PLoS One 13:e0206321. 10.1371/journal.pone.0206321 30439964 PMC6237327

[B14] Derex L. Cho T. H. (2017). Mechanical thrombectomy in acute ischemic stroke. Rev. Neurol. 173, 106–113. 10.1016/j.neurol.2016.06.008 28238346

[B15] Dharap A. Nakka V. P. Vemuganti R. (2012). Effect of focal ischemia on long noncoding RNAs. Stroke 43, 2800–2802. 10.1161/strokeaha.112.669465 22949471 PMC3458128

[B16] Dhiraj D. K. Chrysanthou E. Mallucci G. R. Bushell M. (2013). miRNAs-19b, -29b-2* and -339–5p show an early and sustained up-regulation in ischemic models of stroke. PLoS One 8:e83717. 10.1371/journal.pone.0083717 24376737 PMC3869799

[B17] Dykstra-Aiello C. Jickling G. C. Ander B. P. Shroff N. Zhan X. Liu D.. (2016). Altered expression of long noncoding RNAs in blood after ischemic stroke and proximity to putative stroke risk loci. Stroke 47, 2896–2903. 10.1161/STROKEAHA.116.013869 27834745 PMC5127755

[B12] ENCODE Project Consortium. (2012). An integrated encyclopedia of DNA elements in the human genome. Nature 489, 57–74. 10.1038/nature11247 22955616 PMC3439153

[B18] Geisler S. Coller J. (2013). RNA in unexpected places: long non-coding RNA functions in diverse cellular contexts. Nat. Rev. Mol. Cell Biol. 14, 699–712. 10.1038/nrm3679 24105322 PMC4852478

[B19] Gorup D. Bohacek I. Milicevic T. Pochet R. Mitrecic D. KriΔ J.. (2015). Increased expression and colocalization of GAP43 and CASP3 after brain ischemic lesion in mouse. Neurosci. Lett. 597, 176–182. 10.1016/j.neulet.2015.04.042 25929184

[B20] Guo D. Ma J. Yan L. Li T. Li Z. Han X.. (2017). Down-regulation of lncrna MALAT1 attenuates neuronal cell death through suppressing beclin1-dependent autophagy by regulating Mir-30a in cerebral ischemic stroke. Cell. Physiol. Biochem. 43, 182–194. 10.1159/000480337 28854438

[B21] Gutiérrez M. Merino J. J. Alonso de Leciñana M. Díez-Tejedor E. (2009). Cerebral protection, brain repair, plasticity and cell therapy in ischemic stroke. Cerebrovasc. Dis. 27, 177–186. 10.1159/000200457 19342849

[B22] Hadadha M. Vakili A. Bandegi A. R. (2015). Effect of the inhibition of hydrogen sulfide synthesis on ischemic injury and oxidative stress biomarkers in a transient model of focal cerebral ischemia in rats. J. Stroke Cerebrovasc. Dis. 24, 2676–2684. 10.1016/j.jstrokecerebrovasdis.2015.07.020 26476584

[B23] He W. Wei D. Cai D. Chen S. Li S. Chen W. (2018). Altered long non-coding RNA transcriptomic profiles in ischemic stroke. Hum. Gene Ther. 29, 719–732. 10.1089/hum.2017.064 29284304

[B24] Hu X. L. Olsson T. Johansson I. M. Brannstrom T. Wester P. (2004). Dynamic changes of the anti- and pro-apoptotic proteins Bcl-w, Bcl-2, and Bax with Smac/Diablo mitochondrial release after photothrombotic ring stroke in rats. Eur. J. Neurosci. 20, 1177–1188. 10.1111/j.1460-9568.2004.03554.x 15341589

[B25] Iwata A. Morgan-Stevenson V. Schwartz B. Liu L. Tupper J. Zhu X.. (2010). Extracellular BCL2 proteins are danger-associated molecular patterns that reduce tissue damage in murine models of ischemia-reperfusion injury. PLoS One 5:e9103. 10.1371/journal.pone.0009103 20161703 PMC2816997

[B26] Kopp F. Mendell J. T. (2018). Functional classification and experimental dissection of long noncoding RNAs. Cell 172, 393–407. 10.1016/j.cell.2018.01.011 29373828 PMC5978744

[B28] Li S. Chen L. Zhou X. Li J. Liu J. (2019). miRNA-223–3p and let-7b-3p as potential blood biomarkers associated with the ischemic penumbra in rats. Acta Neurobiol. Exp. 79, 205–216. 10.21307/ane-2019-018 31342956

[B29] Li Z. Li J. Tang N. (2017). Long noncoding RNA Malat1 is a potent autophagy inducer protecting brain microvascular endothelial cells against oxygen-glucose deprivation/reoxygenation-induced injury by sponging miR-26b and upregulating ULK2 expression. Neuroscience 354, 1–10. 10.1016/j.neuroscience.2017.04.017 28433650

[B27] Li J. Tian H. Yang J. Gong Z. (2016). Long noncoding RNAs regulate cell growth, proliferation, and apoptosis. DNA Cell Biol. 35, 459–470. 10.1089/dna.2015.3187 27213978

[B30] Liu B. Cao W. Xue J. (2019). LncRNA ANRIL protects against oxygen and glucose deprivation (OGD)-induced injury in PC-12 cells: potential role in ischaemic stroke. Artif. Cells Nanomed. Biotechnol. 47, 1384–1395. 10.1080/21691401.2019.1596944 31174432

[B31] Liu L. Wang D. Wong K. S. Wang Y. (2011). Stroke and stroke care in China: huge burden, significant workload, and a national priority. Stroke 42, 3651–3654. 10.1161/strokeaha.111.635755 22052510

[B32] Martianov I. Ramadass A. Serra Barros A. Chow N. Akoulitchev A. (2007). Repression of the human dihydrofolate reductase gene by a non-coding interfering transcript. Nature 445, 666–670. 10.1038/nature05519 17237763

[B33] Mercer T. R. Dinger M. E. Mattick J. S. (2009). Long non-coding RNAs: insights into functions. Nat. Rev. Genet. 10, 155–159. 10.1038/nrg2521 19188922

[B34] Merry D. E. Korsmeyer S. J. (1997). Bcl-2 gene family in the nervous system. Annu. Rev. Neurosci. 20, 245–267. 10.1146/annurev.neuro.20.1.245 9056714

[B35] Mikhailov V. Mikhailova M. Pulkrabek D. J. Dong Z. Venkatachalam M. A. Saikumar P. (2001). Bcl-2 prevents Bax oligomerization in the mitochondrial outer membrane. J. Biol. Chem. 276, 18361–18374. 10.1074/jbc.m100655200 11279112

[B36] Minami M. Jin K. L. Li W. Nagayama T. Henshall D. C. Simon R. P. (2000). Bcl-w expression is increased in brain regions affected by focal cerebral ischemia in the rat. Neurosci. Lett. 279, 193–195. 10.1016/s0304-3940(99)00987-8 10688062

[B37] Qureshi I. A. Mattick J. S. Mehler M. F. (2010). Long non-coding RNAs in nervous system function and disease. Brain Res. 1338, 20–35. 10.1016/j.brainres.2010.03.110 20380817 PMC2883659

[B38] Ritz M. F. Curin Y. Mendelowitsch A. Andriantsitohaina R. (2008). Acute treatment with red wine polyphenols protects from ischemia-induced excitotoxicity, energy failure and oxidative stress in rats. Brain Res. 1239, 226–234. 10.1016/j.brainres.2008.08.073 18801346

[B39] Sharp F. R. Jickling G. C. Stamova B. Tian Y. Zhan X. Liu D.. (2011). Molecular markers and mechanisms of stroke: RNA studies of blood in animals and humans. J. Cereb. Blood Flow Metab. 31, 1513–1531. 10.1038/jcbfm.2011.45 21505474 PMC3137473

[B40] Tang Y. Lu A. Aronow B. J. Sharp F. R. (2001). Blood genomic responses differ after stroke, seizures, hypoglycemia, and hypoxia: blood genomic fingerprints of disease. Ann. Neurol. 50, 699–707. 10.1002/ana.10042 11761467

[B41] Tang Y. Nee A. C. Lu A. Ran R. Sharp F. R. (2003). Blood genomic expression profile for neuronal injury. J. Cereb. Blood Flow Metab. 23, 310–319. 10.1097/01.wcb.0000048518.34839.de 12621306

[B42] Tiedt S. Prestel M. Malik R. Schieferdecker N. Duering M. Kautzky V.. (2017). RNA-Seq identifies circulating miR-125a-5p, miR-125b-5p and miR-143–3p as potential biomarkers for acute ischemic stroke. Circ. Res. 121, 970–980. 10.1161/circresaha.117.311572 28724745

[B43] Ulitsky I. Bartel D. P. (2013). lincRNAs: genomics, evolution, and mechanisms. Cell 154, 26–46. 10.1016/j.cell.2013.06.020 23827673 PMC3924787

[B44] van der Worp H. B. van Gijn J. (2007). Clinical practice. Acute ischemic stroke. N. Engl. J. Med. 357, 572–579. 10.1056/NEJMcp072057 17687132

[B45] Wang J. Zhao H. Fan Z. Li G. Ma Q. Tao Z.. (2017). Long noncoding RNA H19 promotes neuroinflammation in ischemic stroke by driving histone deacetylase 1-dependent M1 microglial polarization. Stroke 48, 2211–2221. 10.1161/strokeaha.117.017387 28630232

[B46] Wiessner C. Allegrini P. R. Rupalla K. Sauer D. Oltersdorf T. McGregor A. L.. (1999). Neuron-specific transgene expression of Bcl-XL but not Bcl-2 genes reduced lesion size after permanent middle cerebral artery occlusion in mice. Neurosci. Lett. 268, 119–122. 10.1016/s0304-3940(99)00392-4 10406019

[B47] Writing Group Members Mozaffarian D. Benjamin E. J. Go A. S. Arnett D. K. Blaha M. J.. (2016). Heart disease and stroke statistics-2016 update: a report from the American Heart Association. Circulation 133, e38–e360. 10.1161/CIR.0000000000000350 26673558

[B49] Wu Z. Wu P. Zuo X. Yu N. Qin Y. Xu Q.. (2017). LncRNA-N1LR enhances neuroprotection against ischemic stroke probably by inhibiting p53 phosphorylation. Mol. Neurobiol. 54, 7670–7685. 10.1007/s12035-016-0246-z 27844279

[B48] Wu P. Zuo X. Deng H. Liu X. Liu L. Ji A. (2013). Roles of long noncoding RNAs in brain development, functional diversification and neurodegenerative diseases. Brain Res. Bull. 97, 69–80. 10.1016/j.brainresbull.2013.06.001 23756188

[B50] Yuan M. Tang Y. Zhou C. Liu F. Chen L. Yuan H. (2016). Elevated plasma CaM expression in patients with acute cerebral infarction predicts poor outcomes and is inversely associated with miR-26b expression. Int. J. Neurosci. 126, 408–414. 10.3109/00207454.2015.1020537 26001204

[B51] Zhang L. Luo X. Chen F. Yuan W. Xiao X. Zhang X.. (2018). LncRNA SNHG1 regulates cerebrovascular pathologies as a competing endogenous RNA through HIF-1α/VEGF signaling in ischemic stroke. J. Cell. Biochem. 119, 5460–5472. 10.1002/jcb.26705 29377234

[B52] Zhao M. Wang J. Xi X. Tan N. Zhang L. (2018). SNHG12 promotes angiogenesis following ischemic stroke *via* regulating miR-150/VEGF pathway. Neuroscience 390, 231–240. 10.1016/j.neuroscience.2018.08.029 30193860

[B53] Zhu M. Li N. Luo P. Jing W. Wen X. Liang C.. (2018). Peripheral blood leukocyte expression of lncRNA MIAT and its diagnostic and prognostic value in ischemic stroke. J. Stroke Cerebrovasc. Dis. 27, 326–337. 10.1016/j.jstrokecerebrovasdis.2017.09.009 29030044

[B54] Zhu W. Tian L. Yue X. Liu J. Fu Y. Yan Y. (2019). LncRNA expression profiling of ischemic stroke during the transition from the acute to subacute stage. Front. Neurol. 10:36. 10.3389/fneur.2019.00036 30774621 PMC6367239

