# Supplementary material for: Expression Profile and Potential Functions of Circulating Long Noncoding RNAs in Acute Ischemic Stroke in the Southern Chinese Han Population
Source: Front Mol Neurosci. 2019 Nov 29;12:290. doi: 10.3389/fnmol.2019.00290 (PMC6895137; doi:10.3389/fnmol.2019.00290)

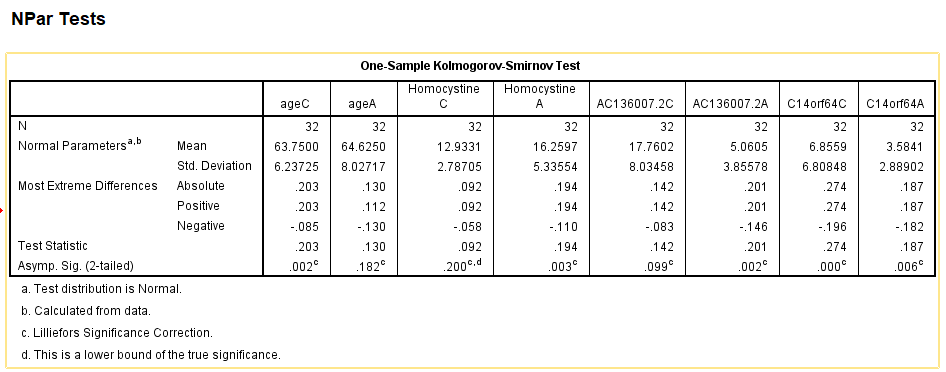


| **Frequencies** | | |
| --- | --- | --- |
|  | group | N |
| age | 1.00 | 32 |
|  | 2.00 | 32 |
|  | Total | 64 |
| Homocystine | 1.00 | 32 |
|  | 2.00 | 32 |
|  | Total | 64 |
| AC136007.2 | 1.00 | 32 |
|  | 2.00 | 32 |
|  | Total | 64 |
| C14orf64 | 1.00 | 32 |
|  | 2.00 | 32 |
|  | Total | 64 |

| **Test Statistics^a^** | | | | | |
| --- | --- | --- | --- | --- | --- |
|  | | age | Homocystine | AC136007.2 | C14orf64 |
| Most Extreme Differences | Absolute | .188 | .375 | .813 | .375 |
|  | Positive | .188 | .375 | .000 | .000 |
|  | Negative | -.156 | .000 | -.813 | -.375 |
| Kolmogorov-Smirnov Z | | .750 | 1.500 | 3.250 | 1.500 |
| Asymp. Sig. (2-tailed) | | .627 | .022 | .000 | .022 |
| a. Grouping Variable: group | | | | | |

**Homocystine**

| **Ranks** | | | | |
| --- | --- | --- | --- | --- |
|  | group | N | Mean Rank | Sum of Ranks |
| homocystine | control | 32 | 26.19 | 838.00 |
|  | acute stroke | 32 | 38.81 | 1242.00 |
|  | Total | 64 |  |  |

| **Test Statistics^a^** | |
| --- | --- |
|  | homocystine |
| Mann-Whitney U | 310.000 |
| Wilcoxon W | 838.000 |
| Z | -2.713 |
| Asymp. Sig. (2-tailed) | .007 |
| a. Grouping Variable: group | |


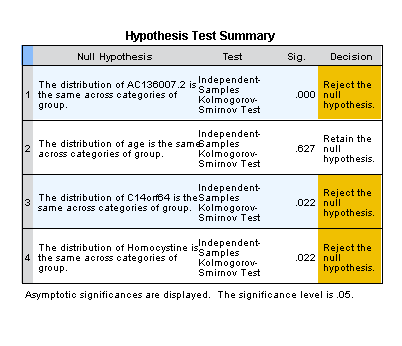


**Homocystine**

| **Ranks** | | | | |
| --- | --- | --- | --- | --- |
|  | group | N | Mean Rank | Sum of Ranks |
| homocystine | control | 32 | 26.19 | 838.00 |
|  | acute stroke | 32 | 38.81 | 1242.00 |
|  | Total | 64 |  |  |

| **Test Statistics^a^** | |
| --- | --- |
|  | homocystine |
| Mann-Whitney U | 310.000 |
| Wilcoxon W | 838.000 |
| Z | -2.713 |
| Asymp. Sig. (2-tailed) | .007 |
| a. Grouping Variable: group | |

| **Frequencies** | | |
| --- | --- | --- |
|  | group | N |
| homocystine | control | 32 |
|  | acute stroke | 32 |
|  | Total | 64 |

| **Test Statistics^a^** | | |
| --- | --- | --- |
|  | | homocystine |
| Most Extreme Differences | Absolute | .375 |
|  | Positive | .375 |
|  | Negative | .000 |
| Kolmogorov-Smirnov Z | | 1.500 |
| Asymp. Sig. (2-tailed) | | .022 |
| a. Grouping Variable: group | | |

**Age**


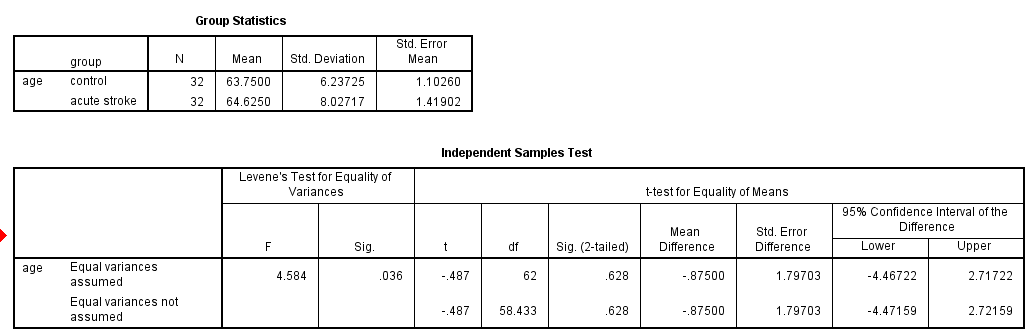


**lncRNA-AC136007.2**


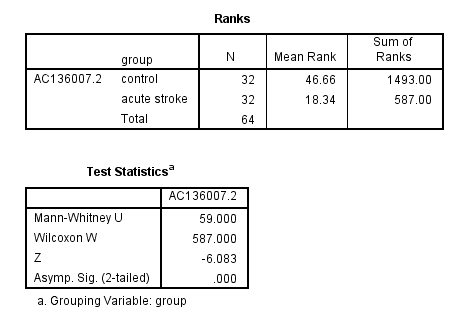


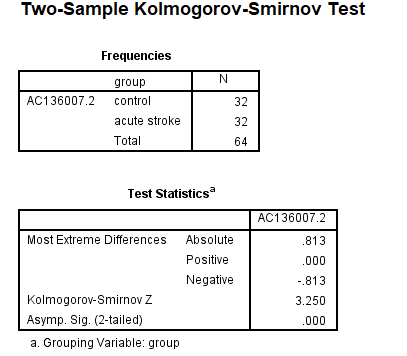


**lncRNA-C14orf64**


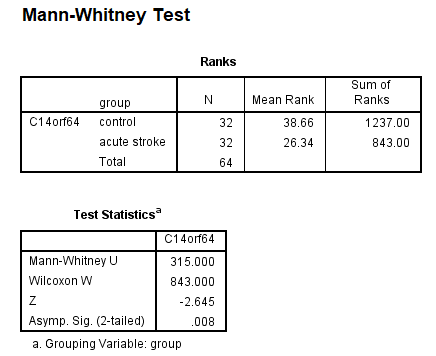


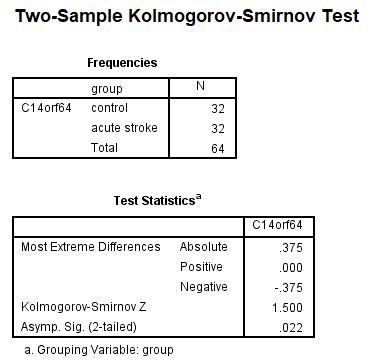


**Gender**


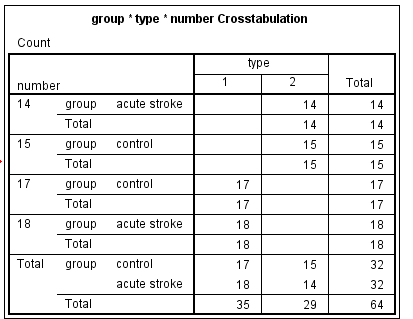


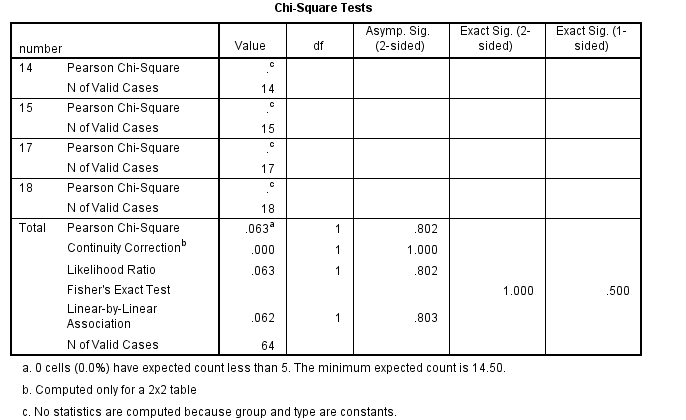


**Hypertension**


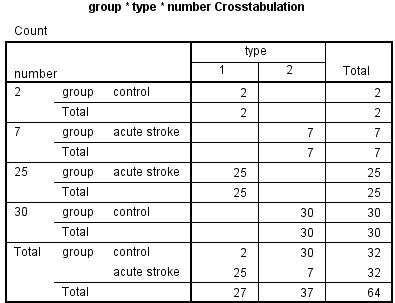


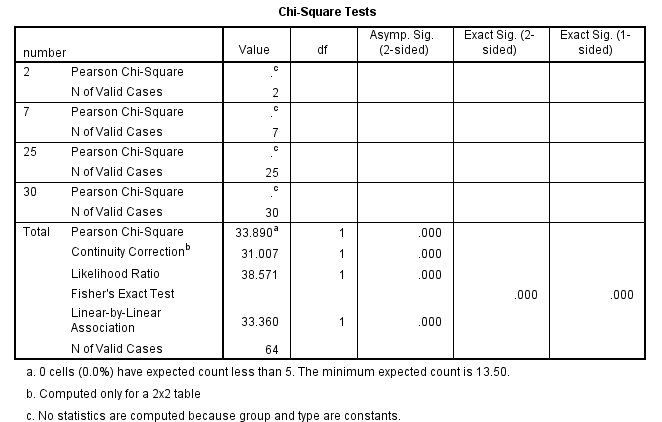


**Diabetes mellitus**


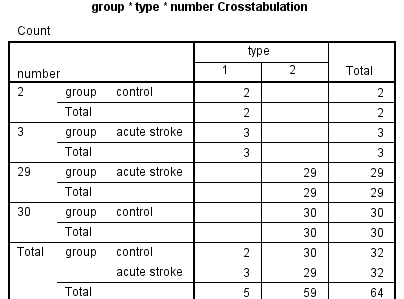


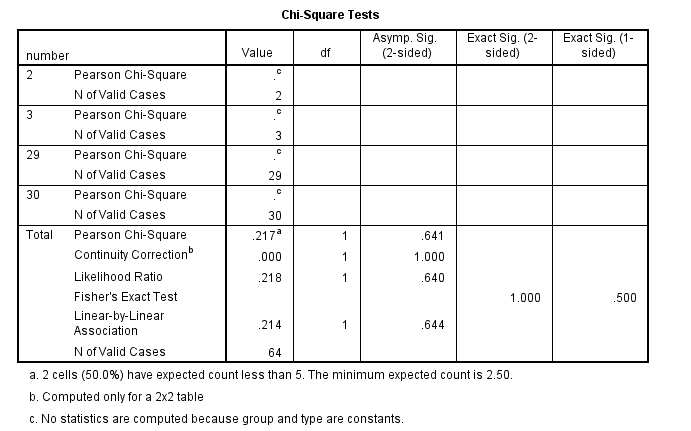


**Hyperlipidemia**


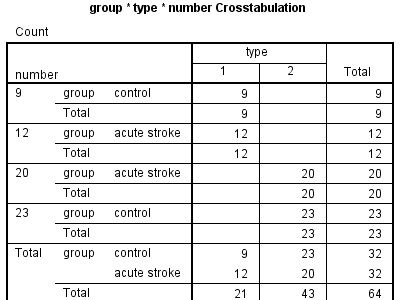


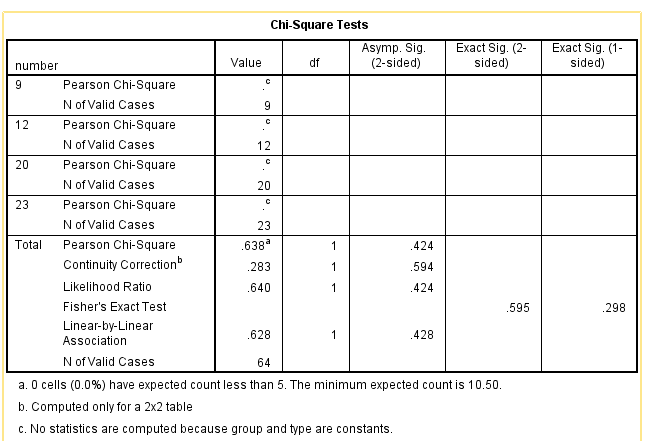

Supplement: TABLE S1 — Results of statistical analysis. [file Table_1.DOCX]
